# Edible Flowers**—**A New Promising Source of Mineral Elements in Human Nutrition

**DOI:** 10.3390/molecules17066672

**Published:** 2012-05-31

**Authors:** Otakar Rop, Jiri Mlcek, Tunde Jurikova, Jarmila Neugebauerova, Jindriska Vabkova

**Affiliations:** 1 Department of Food Technology and Microbiology, Faculty of Technology, Tomas Bata University in Zlin, Namesti T. G. Masaryka 275, CZ-762 72 Zlin, Czech Republic; Email: rop@ft.utb.cz; 2 Department of Natural and Informatics Sciences, Faculty of Central European Studies, Constantine the Philosopher University in Nitra, Drazovska 4, SK-949 74 Nitra, Slovak Republic; Email: tjurikova@ukf.sk; 3 Department of Vegetable Growing and Floriculture, Faculty of Horticulture, Mendel University in Brno, Valticka 337, CZ-691 44 Lednice, Czech Republic; Email: neugebj@zf.mendelu.cz (J.N.); jindriska.vabkova@seznam.cz (J.V.)

**Keywords:** edible flowers, antioxidants, phenolics, flavonoids, mineral elements

## Abstract

On a global scale, people are demanding more attractive and tasty food. Both the quality of foodstuffs and aesthetic aspects contribute to the appearance of consumed meals. The attraction and appeal of individual dishes could be enhanced by edible flowers. New information concerning the composition and nutritional value of edible flowers is also important and represents a sufficient reason for their consumption. The aim of this study is to contribute to the popularization of some selected edible flowers of ornamental plants involving altogether 12 species. The flowers were used to determine their antioxidant capacity, which fluctuated between 4.21 and 6.96 g of ascorbic acid equivalents (AAE)/kg of fresh mass (FM). Correlation coefficients between antioxidant capacity and the contents of total phenolics and flavonoids were r^2^ = 0.9705 and r^2^ = 0.7861, respectively. Moreover, the results were supplemented with new data about the mineral composition of edible flowers (mostly, not found in the available literature). The highest levels of mineral elements were observed in the flowers of species *Chrysanthemum*, *Dianthus *or *Viola*. The most abundant element was potassium, the content of which ranged from 1,842.61 to 3,964.84 mg/kg of FM.

## 1. Introduction

For centuries edible flowers have been an integral part of human nutrition and were already described in detail in ancient literature. In Central Europe for example, fried batter-coated black elder (*Sambucus nigra*) flowers are common, as well as dandelion flowers boiled with sugar (in the past, they were used as ersatz honey). Furthermore, flowers were used as decorations in food prepared for the nobility, especially for feasts and banquets [1]. Nowadays, sales of fresh, top-quality flowers for human consumption are increasing worldwide. These products, suitably packed in bunches, boxes, *etc*. are sold either directly in farm shops or through various specialized outlets [2].

There are several reasons why the interest in edible flowers is continuously increasing. Globalization has contributed not only to a better awareness of consumers but also to the comeback of earlier lifestyles, in which edible flowers played an important role. In China and Japan, edible flowers have been consumed for thousands of years [3,4]. Moreover, new food-processing technologies as well as new logistic methods and quick distribution of cooled and well preserved foodstuffs has enabled us to return to earlier common and widespread food resources. This increasing demand has been and still is associated with efforts of producers and manufacturers of ready-to-cook food to extend and improve their offerings and to introduce new kinds of commodities. Nowadays, edible flowers are used as garnishes and mostly consumed fresh. Nevertheless, they can also be consumed dried, in cocktails (in ice cubes), canned in sugar, preserved in distillates, *etc*. [5].

The contents of common components (proteins, fats, saccharides, vitamins) are not very different from those in other plant organs, e.g., in leaf vegetables [1,6,7,8]. The main criteria for evaluation of edible flower quality are their sensory characteristics, *i.e*., appeal, size, shape, colour, and (above all) taste and aroma [9]. Their colours are predetermined by many chemical compounds but the contents of carotenoids and flavonoids are the most important [10]. A high antioxidant capacity of flowers is mostly correlated just with the level of flavonoids [11]. The main sources of edible flowers are vegetables as well as fruit, medicinal and ornamental plants [12].

Particular species of edible flowers used in the work have different colours and taste—depending on different cultivars: *Antirrhinum majus* (yellow, bitter flavour), *Centaurea cyanus* (blue, vegetal flavour), *Chrysanthemum frutescens* (orange yellow, slightly to very bitter flavour), *Dianthus caryophyllus* (dark pink, slightly bitter flavour), *Fuchsia x hybrida* (reddish and pinkish purple, slightly acidic flavour), *Impatiens walleriana* (pink, sweet flavour), *Rosa odorata* (red, sweet and aromatic flavour), *Tropaeolum majus* (red, sharp and cress-like flavour), *Viola x wittrockiana* (two coloured petals—yellow and violet, sweet flavour), to flowers consisting of only one colour: *Begonia boliviensis* (reddish orange, slightly lemon flavour), *Chrysanthemum parthenium* (white yellow, slightly to very bitter flavour), *Tagetes patula* (orange, bitterish, clove-like flavour). All samples of edible flowers mentioned in the work were non toxic. However, it should be taken into consideration that the limit of the daily intake of the consumption of the flowers of ornamental plants is not yet known [2].

The aim of this study was to determine the mineral elements since there is not so much information on the description of edible flowers in the literature available. In addition, this study was extended and supplemented with a study of the basic antioxidant capacity, the contents of flavonoids and total phenolics of some selected species of edible flowers from ornamental plants.

## 2. Results and Discussion

The results of chemical analyses of the samples of selected edible flowers are provided in Table 1, Table 2, Table 3 and Table 4. The results were expressed as an average of a two-year experiment since there was not a statistically significant difference among the years in any parameter investigated. In the edible flowers studied, the total contents of phenolic substances ranged from 2.53 to 5.11 g of gallic acid/kg of fresh mass (FM). These values were comparable with those found in common kinds of fruit, e.g., plums (3.48–4.95 g of gallic acid/kg of fresh mass [13]), blueberries (3.00–4.89 g of gallic acid/kg of fresh mass [14]), black currants (in average 5.33 g of gallic acid/kg of fresh mass [15]), *etc*. In spite of these values, however, these contents were comparable with or even higher than those normally occurring in other plant products [16], e.g., vegetables such as cabbage (2.36–2.95 of gallic acid/kg of fresh mass [17]), cucumber (in average 0.56 of gallic acid/kg of fresh mass [18]) or onion (in average 1.23 of gallic acid/kg of fresh mass [19]). In this context, it is necessary to say that the composition and nutritional value of each botanical species of edible flowers are quite unique [20]. Unfortunately, there is scarce data about this in the available literature and for that reason this study tries to present new data that could be used when comparing edible flowers with other horticultural crops. In our opinion, these data could be used as a basis for the elaboration of modern food data charts for the food processing industry.

The correlation between TPC and TAC, as determined by means of the DPPH test, was r^2^ = 0.9705; y = 0.8941x − 1.1581 (*p* < 0.0001), and the content of TAC ranged from 4.21 to 6.96 g of AAE/kg of FM. The calculated values indicated that—from the viewpoint of human nutrition—the relationship existing between the total content of phenolic compounds and the antioxidant efficiency of edible flowers was similar to correlations found for fruit (apples, plums and non-traditional kinds of fruits such as Blue Honeysuckle and Cornelian Cherry, *etc*.) by various authors [21,22,23,24]. This fact was already mentioned in literature. So, for example, Miao *et al.* [25] mentioned similar values of correlation in the study about flowers of four different plant species. According to these authors, the TPC values for *Chrysanthemum* spp. were at the level of more than 1 g/kg of FM. However, even within the framework of one botanical species, the values of correlation coefficients for TPC and TAC are mostly lower due to differences in colours of individual cultivars [10].

In flowers, the colour is predetermined by many chemical compounds, of which above all the content of flavonoids is important [2]. In edible flowers, the correlation between TFC and TAC was r^2^ = 0.7861; y = 0.2591x + 0.2141 (*p* < 0.001). As regards the TFC, it ranged from 1.23 to 2.27 g of rutin/kg of FM. Similarly, these high amounts of flavonoids may be one of the factors which influence a high antioxidant capacity of flowers as compared with other plant organs [26]. However, a synthesis of total flavonoids can be conditioned by genetic origin of different edible flower species [27]. This results in a light to white colour of flowers [28], which is associated with lower values of TAC—even within the framework of varieties of one species [24].

Today, consumers and producers pay more and more attention to the quality of foodstuffs and to contents of individual compounds and food components. Minerals have been discussed for a long time. Today, there are many papers that try to popularize lesser known and less common species of horticultural crops. Their authors most frequently emphasize their (often unique) nutritional qualities and technological properties. The content of mineral elements is one of the most essential aspects that influence the use of edible flowers in human nutrition.

**Table 1:** Total phenolic content (TPC) (g of gallic acid/kg of FM), total antioxidant capacity (TAC) (g of ascorbic acid equivalents/kg of FM) and total flavonoid content (TFC) (g of rutin/kg of FM) in 12 species of edible flowers, n = 10.

Species	TPC	TAC	TFC
*Antirrhinum majus*	3.49 ± 0.21 ^a^	5.06 ± 0.24 ^a^	1.78 ± 0.18 ^a^
*Begonia boliviensis*	4.92 ± 0.16 ^b^	6.80 ± 0.29 ^b^	1.84 ± 0.20 ^a^
*Centaurea cyanus*	4.76 ± 0.27 ^b^	6.81 ± 0.26 ^b^	1.81 ± 0.21 ^a^
*Chrysanthemum frutescens*	2.53 ± 0.25 ^c^	4.24 ± 0.30 ^c^	1.23 ± 0.17 ^b^
*Chrysanthemum parthenium*	2.72 ± 0.27 ^c^	4.21 ± 0.31 ^c^	1.29 ± 0.20 ^b^
*Dianthus caryophyllus*	5.28 ± 0.41 ^b^	6.96 ± 0.39 ^b^	2.27 ± 0.20 ^c^
*Fuchsia x hybrida*	3.45 ± 0.30 ^a^	5.20 ± 0.21 ^a^	1.66 ± 0.21 ^ab^
*Impatiens walleriana*	4.85 ± 0.28 ^b^	6.89 ± 0.36 ^b^	1.93 ± 0.18 ^ab^
*Rosa odorata*	5.02 ± 0.34 ^b^	6.85 ± 0.38 ^b^	2.04 ± 0.19 ^ac^
*Tagetes patula*	4.58 ± 0.40 ^b^	6.70 ± 0.37 ^b^	1.90 ± 0.22 ^ac^
*Tropaeolum majus*	3.31 ± 0.29 ^a^	5.12 ± 0.20 ^a^	1.35 ± 0.17 ^b^
*Viola x wittrockiana*	5.11 ± 0.37 ^b^	6.65 ± 0.37 ^b^	1.99 ± 0.23 ^ac^

Different superscripts in each column indicate the significant differences in the mean at *p* < 0.05.

**Table 2:** Dry matter (% w/w) and the content of crude protein (g/kg of FM) in 12 species of edible flowers, n = 10.

Species	Dry matter	Crude protein
*Antirrhinum majus*	12.61 ± 0.11 ^a^	4.85 ± 0.27 ^a^
*Begonia boliviensis*	14.20 ± 0.23 ^b^	2.78 ± 0.35 ^b^
*Centaurea cyanus*	9.74 ± 0.20 ^c^	6.73 ± 0.28 ^c^
*Chrysanthemum frutescens*	9.57 ± 0.16 ^c^	6.85 ± 0.45 ^c^
*Chrysanthemum parthenium*	9.86 ± 0.35 ^c^	6.77 ± 0.26 ^c^
*Dianthus caryophyllus*	11.55 ± 0.18 ^d^	6.89 ± 0.44 ^c^
*Fuchsia x hybrida*	8.37 ± 0.31 ^e^	2.41 ± 0.31 ^b^
*Impatiens walleriana*	14.75 ± 0.44 ^b^	4.60 ± 0.32 ^a^
*Rosa odorata*	10.09 ± 0.23 ^c^	2.66 ± 0.30 ^b^
*Tagetes patula*	9.68 ± 0.34 ^c^	2.95 ± 0.38 ^b^
*Tropaeolum majus*	11.27 ± 0.28 ^d^	4.74 ± 0.19 ^a^
*Viola x wittrockiana*	10.01 ± 0.30 ^c^	6.70 ± 0.21 ^c^

Different superscripts in each column indicate the significant differences in the mean at *p* < 0.05.

**Table 3:** Content of macroelements (mg/kg of FM) in 12 species of edible flowers, n = 10.

Species	Phosphorus	Potassium	Calcium	Magnesium	Sodium
*Antirrhinum majus*	417.62 ± 11.21 ^a^	2,861.83 ± 112.21 ^a^	357.20 ± 10.30 ^a^	172.02 ± 7.29 ^a^	87.74 ± 3.42 ^a^
*Begonia boliviensis*	202.11 ± 14.30 ^b^	1,842.61 ± 94.75 ^b^	348.73 ± 12.46 ^a^	149.53 ± 8.60 ^b^	93.34 ± 3.94 ^a^
*Centaurea cyanus*	534.48 ± 9.85 ^c^	3,568.77 ± 109.62 ^c^	246.18 ± 17.88 ^b^	138.49 ± 5.95 ^b^	74.28 ± 2.05 ^b^
*Chrysanthemum frutescens*	428.36 ± 7.62 ^a^	2,617.24 ± 101.35 ^a^	258.55 ± 21.44 ^b^	105.26 ± 8.32 ^c^	89.10 ± 4.50 ^a^
*Chrysanthemum parthenium*	501.29 ± 8.12 ^d^	3,600.34 ± 102.14 ^c^	341.32 ± 13.07 ^a^	195.17 ± 7.15 ^d^	113.31 ± 3.08 ^c^
*Dianthus caryophyllus*	531.35 ± 7.60 ^c^	3,544.81 ± 100.80 ^c^	491.89 ± 15.25 ^c^	186.55 ± 8.07 ^ad^	114.29 ± 3.17 ^c^
*Fuchsia x hybrida*	215.46 ± 11.12 ^b^	1,967.30 ± 94.35 ^b^	239.10 ± 14.00 ^b^	170.71 ± 9.44 ^a^	125.58 ± 3.84 ^d^
*Impatiens walleriana*	382.73 ± 10.32 ^e^	2,835.25 ± 86.74 ^a^	405.62 ± 17.26 ^d^	203.34 ± 5.08 ^d^	94.29 ± 3.77 ^a^
*Rosa odorata*	225.17 ± 6.18 ^b^	1,969.11 ± 92.10 ^b^	275.15 ± 18.55 ^b^	141.83 ± 6.19 ^b^	76.61 ± 1.97 ^b^
*Tagetes patula*	478.25 ± 9.24 ^f^	3,808.72 ± 98.56 ^cd^	346.85 ± 14.14 ^a^	205.19 ± 9.37 ^d^	114.32 ± 3.61 ^c^
*Tropaeolum majus*	481.31 ± 6.82 ^f^	2,453.39 ± 94.73 ^a^	337.23 ± 18.62 ^a^	149.38 ± 8.57 ^b^	88.52 ± 4.27 ^a^
*Viola x wittrockiana*	514.62 ± 10.32 ^cd^	3,964.84 ± 85.05 ^d^	486.44 ± 24.65 ^c^	190.05 ± 7.21 ^d^	131.97 ± 3.92 ^d^

Different superscripts in each column indicate the significant differences in the mean at *p* < 0.05.

**Table 4:** Content of microelements (mg/kg of FM) in 12 species of edible flowers, n = 10.

Species	Iron	Manganese	Copper	Zinc	Molybdenum
*Antirrhinum majus*	4.38 ± 0.14 ^a^	5.73 ± 0.29 ^a^	1.62 ± 0.08 ^a^	8.89 ± 0.94 ^a^	0.84 ± 0.05 ^a^
*Begonia boliviensis*	2.65 ± 0.21 ^b^	4.35 ± 0.14 ^b^	1.94 ± 0.09 ^b^	4.60 ± 0.57 ^b^	0.62 ± 0.05 ^b^
*Centaurea cyanus*	6.89 ± 0.25 ^c^	2.29 ± 0.29 ^c^	0.89 ± 0.08 ^c^	7.59 ± 1.29 ^a^	0.49 ± 0.05 ^c^
*Chrysanthemum frutescens*	5.15 ± 0.32 ^d^	7.86 ± 0.31 ^d^	2.20 ± 0.07 ^d^	5.49 ± 0.81 ^b^	0.30 ± 0.06 ^d^
*Chrysanthemum parthenium*	5.83 ± 0.15 ^e^	7.33 ± 0.34 ^d^	2.35 ± 0.08 ^d^	5.94 ± 0.89 ^ab^	0.31 ± 0.06 ^d^
*Dianthus caryophyllus*	9.85 ± 0.25 ^f^	7.49 ± 0.25 ^d^	2.88 ± 0.09 ^e^	7.17 ± 1.31 ^a^	0.55 ± 0.05 ^c^
*Fuchsia x hybrida*	8.12 ± 0.24 ^g^	4.17 ± 0.21 ^b^	2.70 ± 0.09 ^e^	11.45 ± 1.24 ^c^	0.71 ± 0.07 ^b^
*Impatiens walleriana*	7.26 ± 0.16 ^c^	6.05 ± 0.27 ^a^	1.31 ± 0.10 ^f^	8.72 ± 1.02 ^a^	0.39 ± 0.06 ^cd^
*Rosa odorata*	3.55 ± 0.18 ^h^	3.44 ± 0.20 ^e^	2.28 ± 0.10 ^df^	4.55 ± 0.80 ^b^	0.64 ± 0.06 ^b^
*Tagetes patula*	8.72 ± 0.24 ^i^	7.86 ± 0.30 ^d^	1.09 ± 0.07 ^g^	13.29 ± 1.12 ^d^	0.37 ± 0.05 ^d^
*Tropaeolum majus*	6.47 ± 0.13 ^j^	5.85 ± 0.24 ^a^	1.17 ± 0.11 ^fg^	9.07 ± 1.27 ^a^	0.29 ± 0.06 ^d^
*Viola x wittrockiana*	7.29 ± 0.19 ^c^	7.93 ± 0.27 ^d^	1.95 ± 0.10 ^bc^	11.52 ± 1.06 ^c^	0.84 ± 0.07 ^a^

Different superscripts in each column indicate the significant differences in the mean at *p* < 0.05.

The results of this study comparing the contents of crude protein and mineral elements in edible flowers are interesting and new. Until now, the results provided here have not been published by other authors. As shown in Table 3, edible flowers are an excellent source of minerals, especially of phosphorus and potassium. The contents of these elements ranged from 202.11 mg/kg to 514.62 mg/kg of FM and from 1,842.61 mg/kg to 3,964.84 mg/kg of FM, respectively. The average content of sodium was 100.28 mg/kg of FM. These values were comparable or higher than those determined in the some kinds of fruit [e.g., pears (in the case of potassium 1,260 mg/kg of fresh mass) or raspberries (in the case of potassium 1,780 mg/kg of fresh mass) or vegetable species, e.g., zucchini (in the case of potassium 1,520 mg/kg of fresh mass) or cucumber (in the case of potassium 1,620 mg/kg of fresh mass)] [6]. Similarly, this fact was observed in the case of microelements. The consumption of crops rich in potassium is recommended for the prevention of cardiovascular or oncogenic diseases [29]. Together with sodium this element is involved in the regulation of osmotic pressure [20]. Minerals make up about 4.7% by weight of the human organism. Most of the minerals are salts containing calcium and phosphorus, as the building blocks of the human skeleton [30]. Generally, macroelements and microelements are the fundamental part of enzyme systems. They serve as the prevention of many diseases and strengthen the human immune system [31]. In real terms, edible flowers are mentioned in association with anti-inflammatory [32], antibacterial [33], antifungal [34] and antiviral effects [35]. Mineral elements can be one of the causes of these effects [2]. The content of crude protein was on average 4.91 g/kg of FM. However, with the exception of cereals, the contents of crude protein are a typical trait of the majority of plant raw materials and in case of edible flowers it was demonstrated that they were similar to fruit or vegetables [6]. In any case, however, edible flowers can be considered an excellent source of minerals in human nutrition.

The major functions of the described mineral elements in the human body are the following—according to the literature source [20]: nitrogen is important for protein synthesis. Phosphorus is found in nucleic acids, ATP, and phospholipids; bone formation; buffers; metabolism of sugars. Potassium is important for nerve and muscle action, protein synthesis or as principal positive ion in cells. Calcium is found in bones and teeth, blood clotting, nerve and muscle action as well as enzyme activation. Magnesium is required by many enzymes; we can also find this element in bones and teeth too. Sodium is important in nerve and muscle action and water balance; it is principal positive ion in tissue fluids.

As regards microelements, iron is found in the active sites of many redox enzymes and electron carriers such as hemoglobin, and myoglobin. Copper has importance in the active site of many redox enzymes and electron carriers, the production of hemoglobin or bone formation. Manganese activates many enzymes. Molybdenum is also found in some enzymes. Cobalt is found in vitamin B_12_. This microelement is important in the formation of red blood cells [20].

On the other hand, the consumption of flowers which are less known or toxic for humans may be dangerous. When consuming flowers picked up freely in Nature, it is always necessary to identify them exactly. The number of flowers which are used for cooking and their soundness (*i.e*., absence of pathogens) may also be limiting factors. It is explicitly recommended not to consume edible flowers of plants originating from non-tested cultivars and florist’s shops because they could be affected by fertilizers and pesticides. Besides toxic effects it is also possible that even the flowers, which are sound, clean and seem to be trouble free, may induce toxic and allergic reactions in people who are sensitive to some of their non-defined components. The amount of eaten flowers can also be a limiting factor. Research in this area will be necessary to find the best species, cultivars and recommended quantities for consumption [2].

## 3. Experimental

Flowers were harvested on the grounds of experimental orchards of Tomas Bata University in Zlin within the period of 2010–2011. These orchards are situated in the south-western part of the White Carpathians near Zlin, Czech Republic. The average altitude is 340 m above sea level, and the mean annual temperature and precipitation are 7.9 °C and 760 mm, respectively. The soil type was classified as the Mesotrophic Cambisol; the value of pH/KCl = 5.58. Agrochemical characteristics of the soil used is given in Table 5 [36]. In the locality and soil described above, the plants were grown in an unheated glasshouse.

**Table 5:** Agrochemical characteristics of the soil used [36].

Mineral element	Content in soil (mg/kg)	Mineral element	Content in soil (mg/kg)
Phosphorus	51.85	Iron	5,360.20
Potassium	156.32	Manganese	551.60
Calcium	3,141.45	Copper	19.71
Magnesium	295.15	Zinc	21.14
Sodium	45.73	Molybdenum	3.12

Flowers were harvested in full ripeness from five randomly chosen plants of each species (cultivar). The degree of edible flower full ripeness was determined on the basis of flower size, opening and colouring [37]. Flowers from each species (cultivar) were mixed together and used for analyses (*i.e*., altogether five per each species).

Flowers of individual species (cultivar) were processed immediately after the harvest (not later than within 24 h). Harvested flowers were pureed in a laboratory grinder SJ500 (MEZOS, Hradec Kralove, Czech Republic) and the average sample was obtained by dividing into quarters. Each parameter was measured in five replications. The results were expressed as the average of a two-year experiment.

The species and cultivars of edible flowers which were analyzed are listed in Table 6.

**Table 6:** Species and cultivars of edible flowers analyzed (modified according to [37,38,39,40]).

Latin name	English name	Cultivar
*Antirrhinum majus*	Snapdragon	Zluty Kral
*Begonia boliviensis*	Bolivian Begonia	Bonfire
*Centaurea cyanus*	Cornflower	Modracek
*Chrysanthemum frutescens*	Marguerite Daisy	Silver Leaf
*Chrysanthemum parthenium*	Feverfew	Roya
*Dianthus caryophyllus*	Clove Pink	Picotee
*Fuchsia x hybrida*	Fuchsia	Autumnale
*Impatiens walleriana*	Busy Lizzy	Rockapulco Purple
*Rosa odorata*	Tea Rose	Ilona
*Tagetes patula*	French Marigold	Bolero
*Tropaeolum majus*	Nasturtium	Tom Pouce
*Viola x wittrockiana*	Pansy	Fancy

### 3.1. Extraction of Samples

The extraction was performed according to the method described by Kim *et al.* [41] and modified according to Barros *et al.* [42], using the following procedure: a fresh sample (10 g) was homogenized for 10 s in methanol (100 mL). The resulting paste was placed into Erlenmeyer flasks (120 mL) and allowed to stand in a water bath at a temperature of +25 °C for a period of 24 h. The residue was then extracted with two additional portions of methanol. The combined methanolic extracts were evaporated to dryness at +40 °C [rotary evaporator R-215 (Buchi Ltd., Oldham, UK)] and redissolved in methanol at a concentration of 100 mg/mL, and stored at +4 °C for further use.

### 3.2. Total Phenolic Content (TPC) Assay

To measure total contents of phenolic substances, the sample (0.5 mL of methanolic extract) was taken and diluted with water in a 50-mL volumetric flask. Thereafter, Folin-Ciocalteau reagent (2.5 mL) and a 20% solution of Na_2_CO_3_ (7.5 mL) were added. The resulting absorbance was measured on a LIBRA S6 spectrophotometer (Biochrom Ltd., Cambridge, UK) at a wavelength of 765 nm against a blank sample, which was used as reference. The results were expressed as g of gallic acid (GAE)/kg of fresh mass (FM) [41].

### 3.3. Antioxidant Capacity (TAC) by the DPPH Test Assay

The 2,2-diphenyl-1-picrylhydrazyl (DPPH) test was conducted according to the method of Brand-Williams *et al.* [43] with some modifications [44]. The stock solution was prepared by dissolving DPPH (24 mg) with methanol (100 mL) and then stored at −20 °C until needed. The working solution was obtained by mixing stock solution (10 mL) with methanol (45 mL) to obtain an absorbance of 1.1 ± 0.02 units at 515 nm using the LIBRA S6 spectrophotometer. Flower extracts (150 µL) were allowed to react with the DPPH solution (2,850 µL) for one hour in the dark. Then the absorbance was taken at 515 nm. Antioxidant capacity was calculated as a decrease in the absorbance value using the formula: 


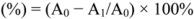


where A_0_ is the absorbance of the control (without the sample) and A_1_ is the absorbance of the mixture containing the sample. The absorbance results were converted using a calibration curve of the standard and expressed as g of ascorbic acid equivalents (AAE)/kg of FM [23].

### 3.4. Total Flavonoid Content (TFC) Assay

The total flavonoid content (TFC) was determined using the sample (0.3 mL), 30% ethanol (3.4 mL), NaNO_2_ (0.1 mL, 0.5 mol/L) and AlCl_3_·6H_2_O (0.15 mL, 0.3 mol/L) following Park *et al.* [45]. The mixture was measured at the wavelength of 506 nm using the LIBRA S6 spectrophotometer. The total flavonoid concentration was calculated from a calibration curve using rutin as the standard. The results were expressed in g/kg of FM.

### 3.5. Dry Matter (DM) and Mineral Content Assay

The dry matter and mineral content were measured by modified methods described in [46,47]. The sample was dried to a constant weight in a VENTICELL 111 laboratory oven (BMT, Brno, Czech Republic) at a temperature of 105 °C ± 2 °C. The dry matter was expressed as percentage of w/w. A portion of homogenized dry matter (DM, 1 g) [SJ500 laboratory grinder (MEZOS, Hradec Kralove, Czech Republic)]—the size of particles up to 1 mm—was thereafter mineralized in a mixture of concentrated sulphuric acid and 30% hydrogen peroxide in digestion tubes placed in a heating block digester (BLOC DIGEST M 24 apparatus, JP/Selecta, Abrera, Spain). The mineralized samples were quantitatively transferred into a 250-mL volumetric flask and its volume was refilled to the volume with re-distilled water. The mineralizate was measured in a PHILIPS PU 9200X atomic absorption spectrometer (Philips, Eindhoven, The Netherlands). The content of phosphorus in the mineralizate was measured by using the LIBRA S6 spectrophotometer. The mineralizate (10 mL) was pipetted into a 100-mL volumetric flask, ammonium-vanadomolybdate reagent (10 mL) was added; the flask was refilled to the volume with re-distilled water and the sample was measured at a wavelength of 410 nm. As a standard stock solution KH_2_PO_4_ was used. The amount of minerals was expressed as mg/kg of FM. The content of total nitrogen was determined according to Kjeldahl. The KJELTEC TM 2300 apparatus (Foss, Hillerod, Denmark) with automatic distillation and approved colorimetric titration with a 100 mL tube was used. The content of nitrogen was multiplied by the coefficient 6.25 and expressed as crude protein in g/kg of FM.

### 3.6. Statistical Analysis

The data obtained were analyzed statistically by the analysis of variance (ANOVA) and Tukey’s multiple range test for comparison of means [48]. Correlation functions were calculated using the Unistat, v. 5.1 statistical package and Office Excel^®^ Microsoft 2010.

## 4. Conclusions

The results obtained explicitly indicate the value of edible flowers. Edible flowers can be used as raw material for the production of various food products, in gastronomy, *etc*. This paper provides data about the high content of mineral elements in 12 edible flower species, which is higher than in most fruit or vegetable species. A high nutritional value, antioxidant capacity and attractive appearance predetermine edible flowers to be a new and promising foodstuff species for a wider use in human nutrition. Education of the public and the promotion of edible flowers are also very important and this study was conducted just for that reason. The obtained results should contribute to the popularization of edible flowers as a new and prospective source for the food industry, gastronomy as well as a promising object of human nutrition.
